# Renal Findings in Patients with Thalassemia at Abdominal Ultrasound: Should We Still Talk about “Incidentalomas”? Results of a Long-Term Follow-Up

**DOI:** 10.3390/diagnostics14182047

**Published:** 2024-09-15

**Authors:** Carmina Fatigati, Antonella Meloni, Silvia Costantini, Anna Spasiano, Flora Ascione, Filippo Cademartiri, Paolo Ricchi

**Affiliations:** 1 Rare Red Blood Cells Diseases Unit, Azienda Ospedaliera di Rilievo Nazionale “A. Cardarelli”, 80131 Naples, Italy; fatigatimelania@gmail.com (C.F.); silvia.costantini@aocardarelli.it (S.C.); spasiano.anna@tiscali.it (A.S.); 2 Bioengineering Unit, Fondazione G. Monasterio CNR-Regione Toscana, 56124 Pisa, Italy; antonella.meloni@ftgm.it; 3 Department of Radiology, Fondazione G. Monasterio CNR-Regione Toscana, 56124 Pisa, Italy; fcademartiri@ftgm.it; 4 Direzione Sanitaria, Azienda Ospedaliera di Rilievo Nazionale “A. Cardarelli”, 80131 Naples, Italy; flora.ascione@aocardarelli.it

**Keywords:** thalassemia, renal stones, renal cysts, renal-cell carcinoma

## Abstract

We retrospectively collected all ultrasound imaging data of our thalassemia patients over a period of 10 years with the aim of assessing the prevalence and the risk factors of renal stones and cysts. Moreover, we assessed the incidence of renal-cell carcinoma (RCC) among thalassemia patients (133 with thalassemia major (TM) and 157 with thalassemia intermedia (TI)) and its association with demographic and clinical findings. Renal stones were detected in 15.2% of patients. In the multivariable Cox regression analysis, the independent predictors were blood consumption, splenectomy, and proteinuria. Renal cysts were detected in 18.4% of patients. In the multivariable analysis, age emerged as the only independent predictor. After the first detection, 35% of the patients showed changes in the number, size, or grading of renal cysts. During the study period, the crude incidence rate of RCC was 75.9 cases per 100,000 person-years. The most frequent histological subtype (80%) included clear-cell RCC. In total, 80% of patients with RCC had TM and all were positive for hepatitis C virus antibodies. Thalassemia patients are significantly affected by asymptomatic renal diseases such as stones, cysts, and cancer, suggesting the need for regular screening by imaging.

## 1. Introduction

Thalassemia syndromes represent the most common inherited monogenic disorders worldwide, and in Italy they constitute an important public-health challenge, affecting about 7000 people [1]. Thalassemias are heterogeneous disorders caused by reduced or absent alpha or beta globin synthesis, which are major components of adult hemoglobin A (HbA, α2β2), resulting in an imbalance of the globin chains [2,3]. The hematological and clinical features of thalassemia disease encompass mild and clinically overt conditions, including thalassemia minor or asymptomatic carrier, thalassemia intermedia (TI), and thalassemia major (TM) [4]. Patients with thalassemia have a great tendency to develop complications of the disease, particularly in the absence of optimal management in specialized centers [5,6,7], where they are more likely to receive appropriate and targeted chelation therapy for the prevention/removal of iron overload and therapy for the management of complications such as cardiac diseases and endocrinopathies.

There is mounting evidence that the kidney is a novel target of the disease [8,9]. Although the exact underlying pathophysiologic mechanisms are not yet fully identified, chronic anemia, hypoxia, and iron overload but also iron chelation have been indicated as key components in the development of renal damage, which often shows features of tubular and glomerular dysfunction [10]. In fact, anemia decreases the systemic vascular resistance and generates hyperdynamic circulation in glomeruli [11]; the consequent rise in hydrostatic pressure within the glomerular capillaries determines glomerular hyperfiltration [12], which is responsible for arterial wall stretching and ultimately leads to vascular damage and sclerosis [13]. Anemia is also responsible for hypoxic damage [14], particularly involving cells in the proximal tubule because of their high rate of metabolic activity, and thus can lead to cellular apoptosis [15,16]. In β-TM patients, a link between proximal tubular dysfunction and intracellular iron deposition has been previously reported [12,17], as iron overload causes tubular oxidative stress, leading to tubular lipid peroxidation and subsequent cell injury/death, and theoretically also to cancer [18,19]. Experimental research using an animal model of β-TM subjected to prolonged iron loading revealed that rats accumulated iron in glomeruli and in the proximal but not in distal tubules, with signs of marked glomerulosclerosis, tubular atrophy, and interstitial fibrosis [20]. On the other hand, iron may impact on renal arterial vasodilation, which is a cofactor for renal prostaglandin synthesis [21], and therefore, in the case of “relative iron depletion”, iron chelators could be responsible for the reduced glomerular filtration (GFR) observed with deferoxamine and deferasirox.

Tubular injury is the cause of many urine abnormalities seen in β-TM patients, such as an increase in proteinuria, albuminuria, calciuria, phosphaturia, uricosuria, and β2-microglobulin [22,23]. In a recent metabolomic study, 60% of patients exhibited hypercalciuria and many individuals had impaired urine concentration. Several amino acids, N-methyl compounds, and organic acids were overexcreted in the urine of thalassemic patients compared with controls [24]. Such a raised excretion in some urinary solutes in thalassemia patients is likely at the basis of the high prevalence of nephrolithiasis seen in this population [25,26].

Abdominal ultrasonography (US) is an important component in the monitoring of thalassemia patients, due to the frequent need to both assess the size and structure of abdominal organs and to screen patients affected by chronic hepatitis C virus (HCV) infection [27,28]. As a result of the growth in imaging utilization in patients with thalassemia, a high prevalence and/or incidence of nephrolithiasis, renal cysts, and tumors were also described in this population [26,29]. In most cases, these reports were from asymptomatic patients and therefore were frequently classified as incidentalomas as in the case of non-thalassemic populations. 

We retrospectively collected all US imaging data of our thalassemia patients over a period of 10 years with the aim of assessing the prevalence and risk factors of renal stones and cysts. Moreover, this study aimed at assessing the lifetime incidence of renal-cell carcinoma (RCC) among thalassemia patients and its association with the type of thalassemia and demographic and clinical findings. 

## 2. Materials and Methods

### 2.1. Study Design and Data Collection

This observational retrospective study was conducted through a review of medical records of all thalassemia patients referred at the AORN Cardarelli center in Naples, Italy. Patients with hemoglobin S/β-thalassemia were not included in the analysis.

For each patient, demographic data (gender and date of birth) and disease-related data, including type of thalassemia (intermedia or major) and genotype, were extracted. 

Follow-up was conducted until 15 June 2024 (study end date). For patients who were still alive, this date was taken as the censoring date. For patients who transferred to a different thalassemia center, the date of transferring was taken as the censoring date.

Starting from 2014, abdominal US was systematically and routinely performed in all patients. The year 2014 represented the baseline time point for the first part of the study, which assessed the risk factors for the development of renal cysts and stones. The electronic medical records of all thalassemia patients were retrospectively reviewed for clinical data, laboratory exams, and magnetic resonance imaging (MRI) data at baseline. Moreover, for those patients with cysts/stones, clinical, laboratory, and MRI characteristics at the time of cyst/stone detection were assessed. For each considered end-point, the date of its first detection was employed as the censoring date.

Data about the onset, characteristics, and evolution of renal-cell carcinoma was collected from birth to the end of follow-up. Moreover, clinical findings, laboratory data, and hepatic iron levels by MRI at the time of RCC detection were also extracted. 

The study complied with the Declaration of Helsinki. All patients gave written informed consent to the protocol. The study was approved by the Ethics Committee of the Cardarelli Hospital, Naples (protocol code: 234; approval date: 21 June 2018).

### 2.2. Assessment of Renal Stones, Cysts, and Tumors

The diagnosis of stones and clinical benign cystic lesions was made by a team of expert ultrasonographists on routine ultrasonography. Contrast-enhanced ultrasound (CEUS) was used to further evaluate indeterminate renal lesions [30,31]. In suspected malignant cases, following urologist consultation, computed tomography (CT) scan or MRI was performed [32].

### 2.3. Laboratory Investigations 

Blood was collected by venipuncture, allowed to clot, and then centrifuged to obtain serum samples. Serum ferritin, hemoglobin (pre-transfusion for regularly transfused patients), soluble transferrin receptor-1, creatinine, and uric acid were determined by commercially available kits. 

A serum ferritin level ≥1000 ng/mL indicated significant body iron burden [33]. The normal reference ranges for soluble transferrin receptor-1 were 0.76–1.76 mg/L. Hyperuricemia was defined as a serum uric acid level >7.0 mg/dL in men and >5.7 mg/dL in women [34]. The normal level of serum creatinine was 0.7–1.3 mg/dL for men and 0.6–1.1 mg/dL for women.

### 2.4. Iron Overload Assessment

Hepatic iron overload assessment was performed using the T2 * technique, implemented on clinical 1.5T MRI scanners [35]. A single mid-transverse hepatic slice was obtained at nine echo times (TEs) in a single end-expiratory breath-hold [36]. A custom-written, previously validated software program (HIPPO MIOT^®^ Version 2.0, Consiglio Nazionale delle Ricerche and Fondazione Toscana Gabriele Monasterio, Pisa, Italy) was used for image analysis. The T2* value was calculated in a circular region of interest (ROI) of fixed dimensions, placed in a homogeneous area of parenchyma free from large blood vessels [36]. The calibration curve introduced by Wood et al. was employed to convert liver T2* values into liver iron concentration (LIC) values [37]. A LIC ≥ 3 mg/g/dw was considered indicative of hepatic iron load [38].

### 2.5. Statistical Analysis

All data were analyzed using SPSS version 27.0 (IBM Corp, Armonk, NY, USA) and JMP version 17.0 (SAS Institute, Cary, NC, USA) statistical packages. 

Descriptive statistics were used to characterize the study population. Continuous variables were described as mean ± standard deviation (SD) and categorical variables as frequencies and percentages.

The Kolmogorov–Smirnov test was employed to assess the normality of distribution of the continuous variables.

Comparison between the two groups was made using an independent-samples *t*-test for continuous values with normal distribution, a Mann–Whitney *U* test for continuous values with non-normal distribution, and χ2 testing for categorical data.

For continuous variables, the difference between values at two different time points (baseline and the detection of renal cysts or stones) was analyzed using Student’s *t*-test for paired data or the Wilcoxon signed-rank test.

The Cox proportional hazard model was used to explore the potential association between the prognostic variables of interest and the study’s outcome (renal cysts or stones). Each prognostic variable was first tested using univariate analysis, and those variables achieving statistical significance were included in the multivariate model. Those variables that did not significantly improve the model’s performance were discarded from the final analysis. The results were presented as hazard ratios (HRs) with 95% confidence intervals (CIs).

The cumulative incidence was used to calculate the absolute risk of a renal complication over different periods of time. It was estimated using the Kaplan–Meier product-limit method without accounting for competing risk events. Differences between the groups were tested using the log-rank test.

The incidence rate was calculated by dividing the number of events by the number of person-years.

A 2-tailed probability value of *p* < 0.05 was used as the criterion for statistical significance in all tests.

## 3. Results

### 3.1. Study Population

We considered 290 patients: 133 with TM and 157 with TI. The percentage of female patients was 55.6% in the TM cohort and 57.3% in the TI cohort (*p* = 0.773).

At the study end date (15 June 2024), 256 (88.3%) patients were still alive and followed at our center, 17 (5.9%) patients had died, and 17 (5.9%) were transferred to a different center.

The mean age at the end of follow-up was 45.63 ± 13.72 years. TI patients were significantly older than TM patients (47.82 ± 15.51 years vs. 43.05 ± 10.73 years; *p* = 0.020).

All TM patients were uniformly treated with regular transfusions of packed red blood cells since early childhood and iron chelation therapy. Among the TI patients, 116 (73.9%) were transfusion-naive or had received sporadic blood transfusions, while the remaining 41 (26.1%) followed a regular transfusion regimen (>4 transfusions per year), started in late childhood or adulthood (mean age: 15.57 ± 16.82 years).

Forty-five (15.5%) patients had joined our thalassemia center after 2015 and, due to the absence of the hematochemical data concerning 2014 and of the periodic echography exams, they were not included in the group of patients considered to assess the predictors of renal cysts and stones.

### 3.2. Renal Stones

#### 3.2.1. Development of Renal Stones

Out of the considered 245 patients, 14 (5.7%) showed nephrolithiasis at baseline (2014) and were excluded from this analysis. Table 1 summarizes the baseline demographic, clinical, and MRI features of the 231 considered thalassemia patients. 

The mean follow-up time was 105.39 ± 29.17 months (median = 120.02 months). Renal stones were detected in 35 (15.2%) patients. Among them, 10 (28.6%) patients had typical symptoms and 4 (11.4%) patients received appropriate therapy. 

**Figure 1:**
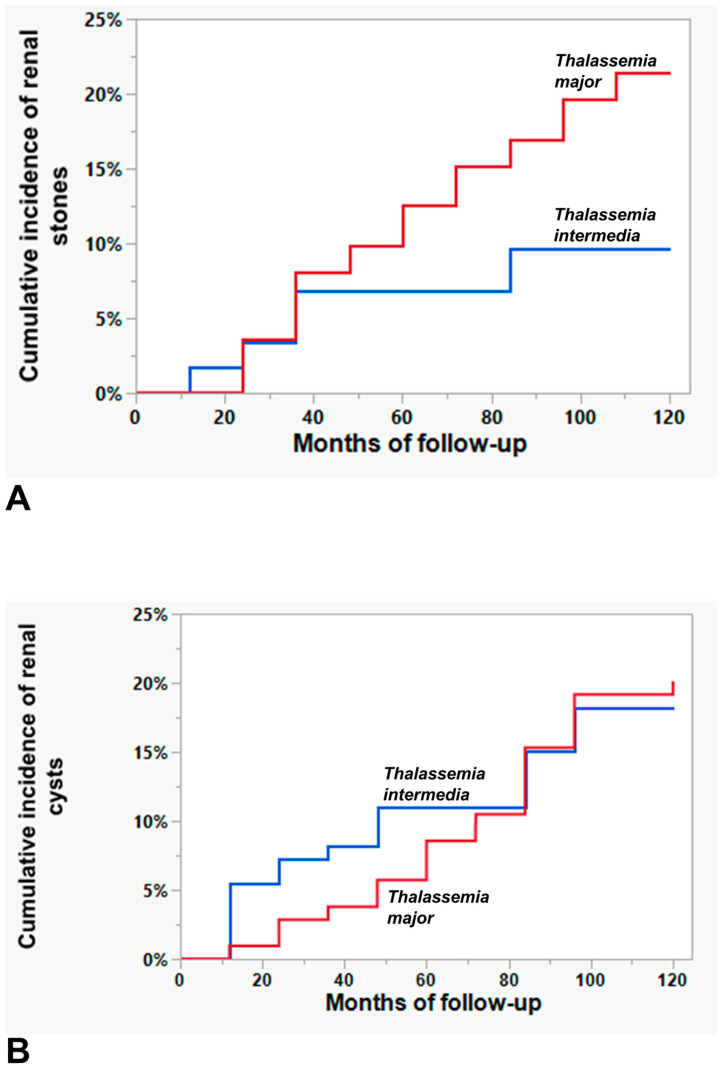
Cumulative incidence or renal stones (**A**) and renal cysts (**B**) in thalassemia major patients (red line) compared to that in thalassemia intermedia patients (blue line).

The prevalence of renal stones was significantly higher in TM patients than in TI patients (21.4% vs. 9.2%; *p* = 0.010). The cumulative incidence of renal stones during the follow-up period was 21.43% (95% CI = 14.79–29.99%) for TM patients and 9.61% (95% CI = 5.59–16.53%) for TI patients, with a significant difference between the two patient groups (*p* = 0.019) (Figure 1A).

The mean time from the baseline to the detection of renal cysts was 54.52 ± 29.01 months. 

In patients with renal stones, the serum levels of ferritin, transferrin receptor-1, creatinine, and uric acid at the time of diagnosis were comparable to the values detected at baseline (*p* > 0.05).

#### 3.2.2. Comparison of Baseline Data between Patients without and with Renal Stones

Table 1 presents the comparison of baseline characteristics between patients who remained free of renal stones and patients who developed renal stones during the study. No significant difference was detected in terms of gender, age, presence of hepatitis C virus (HCV) infection, frequency of diabetes or hypertension, serum levels of ferritin, hemoglobin, and uric acid, or MRI LIC values. Compared to patients free of renal stones, patients with renal stones were more frequently affected by TM than by TI and had a significantly higher annual blood consumption. In the subgroup of TI patients, the frequency of renal stones was significantly higher among regularly transfused patients compared to non-transfused patients, but the difference was not significant (45.5% vs. 29.6%; *p* = 0.280). Moreover, patients with renal stones were more frequently splenectomized and showed a significantly lower serum transferrin receptor-1 level and significantly higher serum creatinine levels and proteinuria.

#### 3.2.3. Prediction of Renal Stones

Table 2 shows the results of the univariate Cox regression analysis for the prediction of renal stones. Being affected by TM, annual blood consumption, splenectomy, and proteinuria emerged as significant univariate prognosticators of renal stones. In the multivariable analysis, the independent predictors were blood consumption (HR = 2.15, 95% CI = 1.46–3.17; *p* < 0.0001), splenectomy (HR = 2.29, 95% CI = 1.14–4.61; *p* = 0.019), and proteinuria (HR = 1.01, 95% CI = 1.00–1.01; *p* = 0.010).

### 3.3. Renal Cysts

#### 3.3.1. Development of Renal Cysts

Out of the considered 245 patients, 28 (11.4%) had a renal cyst at baseline (year 2014) and were excluded from this analysis. Table 3 summarizes the baseline demographic, clinical, and MRI features of the 217 considered thalassemia patients. 

The mean follow-up time was 103.29 ± 31.08 months (median = 120.02 months). Renal cysts were detected in 40 (18.4%) patients. Of these, 16 (40%) patients had only one cyst at first detection, while the remaining 24 (60.0%) patients presented with two or more cysts. Thirty-eight (95.0%) patients had simple renal cysts (Bosniak I) and two (5.0%) patients had minimally complex cysts (Bosniak II). The mean maximum diameter of the cysts was 14.42 ± 9.65 mm.

The prevalence of renal cysts was comparable between TM and TI patients (19.8% vs. 17.1%; *p* = 0.609). The cumulative incidence of renal cysts during the follow-up period was 20.12% (95% CI = 13.50–28.91%) for TM patients and 18.21% (95% CI = 11.89–26.84%) for TI patients. There was no a significant difference between the two patient groups (*p* = 0.835) (Figure 1B).

The mean time from the baseline to the detection of renal cysts was 59.10 ± 32.54 months. 

In patients with renal cysts, the serum levels of ferritin, transferrin receptor-1, creatinine, and uric acid at the time of diagnosis were comparable to the values detected at baseline (*p* > 0.05).

#### 3.3.2. Comparison of Baseline Data between Patients without and with Renal Cysts

Table 3 presents the comparison of baseline characteristics between patients who remained free of renal cysts and patients who developed renal cysts during the study. No significant difference was detected in terms of gender, type of thalassemia, frequency of splenectomy, presence of HCV infection, frequency of diabetes, serum levels of hemoglobin, ferritin, and uric acid, or MRI LIC values. Compared to patients free of renal cysts, patients with renal cysts were significantly older and showed significantly higher body mass index, serum creatine levels, and proteinuria.

#### 3.3.3. Prediction of Renal Cysts

Table 4 shows the results of the univariate Cox regression analysis for the prediction of renal cysts. Age, hypertension, and body mass index emerged as significant univariate prognosticators of renal cysts. In the multivariable analysis, age emerged as the only independent predictor.

#### 3.3.4. Evolution of Renal Cysts

After the first detection, the 40 patients with renal cysts underwent other echography exams to assess their evolution. During a mean observation time of 4.80 ± 2.65 years, the following changes were detected:

– Twenty-six (65.0%) patients: no change in cyst size or grading and no newly developed cysts;
– Six (15.0%) patients: increase in cyst size and no newly developed cysts;
– Two (5.0%) patients: increase in cyst size and grading (from Bosniak I to Bosniak III for one patient and from Bosniak I to Bosniak II for the other patient) and no newly developed cysts;
– Three (7.5%) patients: increase in cyst size and newly developed cysts;
– Two (5.0%) patients: increase in cyst grading (from Bosniak I to Bosniak II) and newly developed cysts;
– One (2.5%) patient: increase in cyst size and grading (from Bosniak I to Boniak II) and newly developed cysts.

Patients with stable cysts and patients showing changes in the number, size, or grading of renal cysts were comparable in terms of baseline serum levels of ferritin, hemoglobin, transferrin receptor-1, creatinine, and uric acid, baseline proteinuria, and baseline MRI LIC values (*p* > 0.05 for all comparisons), while patients showing changes in the number, size, or grading of renal cysts were significantly older (48.22 ± 14.06 years vs. 36.42 ± 9.28 years; *p* = 0.011) and showed significantly higher baseline body mass index (25.65 ± 3.66 kg/m^2^ vs. 23.32 ± 2.61 kg/m^2^; *p* = 0.040) and frequency of hypertension (28.6% vs. 0.0%; *p* = 0.011).

### 3.4. Renal-Cell Carcinoma

#### 3.4.1. RCC: Prevalence and Incidence

During the period of observation, RCC was detected in 10 patients, leading to a global prevalence of 3.4%. The prevalence of RCC was significantly higher in TM patients than in TI patients (6.0% vs. 1.3%; *p* = 0.048).

**Figure 2:**
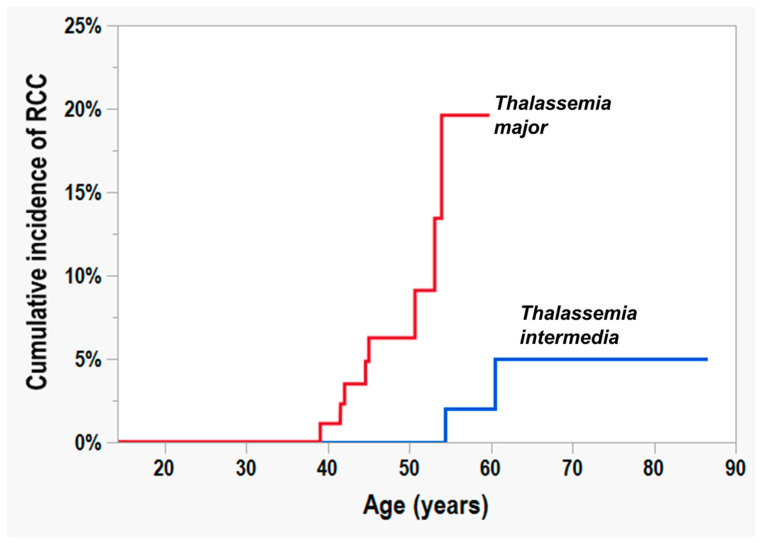
Cumulative incidence or renal-cell carcinoma in thalassemia major patients (red line) compared to that in thalassemia intermedia patients (blue line) (*p* = 0.0007).

The cumulative incidence of RCC in the whole study population was 0.49% (95% CI = 0.07–3.43%) at 40 years of age, 2.79% (95% CI = 1.16–6.56%) at 45 and 50 years of age, 8.01% (95% CI = 3.94–15.60%) at 55 years of age, and 10.80% (95% CI = 5.21–21.05%) at 60 years of age. Among TM patients, the cumulative incidence of RCC was 1.09% (95% CI = 0.15–7.39%) at 40 years of age, 6.24% (95% CI = 2.61–14.19%) at 45 and 50 years of age, and 19.59% (95% CI = 8.36–39.45%) from 55 years of age. Among TI patients, the cumulative incidence of RCC was 2.00% (95% CI = 0.28–12.88%) starting from 55 years of age and 4.97% (95% CI = 1.21–18.26%) at 60 years of age. Figure 2 shows the cumulative incidence of RCC in TM and TM patients. There was a significant difference between the two patient groups (*p* = 0.0007).

The crude incidence rate of RCC was 75.9 cases per 100,000 person-years.

#### 3.4.2. Characteristics of Patients Diagnosed with RCC

Table 5 reports the general characteristics of the patients at the time of diagnosis of RCC. 

The most frequent histological subtype (8/10 patients = 80%) was clear-cell RCC. One patient had chromophobe RCC, while one patient had a rare RCC subtype: mucinous tubular and spindle cell. The right kidney was more frequently involved (80% of the patients). In three (30%) patients, there had been a progression from simple renal cysts (Bosniak I) to renal-cell carcinoma. 

All patients were asymptomatic.

In total, 80% of the patients had TM. The mean age at diagnosis was 48.43 ± 6.97 years and only one patient was under 40 years. All patients were positive for HCV antibodies. One patient had chronic HCV infection while the remaining nine patients had eradicated the virus spontaneously (N = 5) or after the treatment with antiviral therapy (N = 4). Only two patients were smokers. Iron overload indices were available for nine patients. Of these, seven (77.8%) showed hepatic iron overload and/or a ferritin level ≥1000 ng/mL.

All patients for whom the measurement was available showed increased serum transferrin receptor-1 while no patient showed hyperuricemia or an increased serum creatinine. 

At the study end date, 7 of the 10 patients diagnosed with RCC were still alive. The mean survival time for these patients was 66.57 ± 74.77 months (range: 1–204 months). Two deaths were due to carcinoma (renal and hepatic) while one patient died 90 months after RCC diagnosis due to coronavirus disease 2019 (COVID-19).

## 4. Discussion

Renal dysfunction is a frequent and emerging complication in patients suffering from thalassemia. The increasing life expectancy of patients with thalassemia and the tendency (even higher with respect to the general population) to develop complications and comorbidities prompted us to accurately evaluate radiological renal findings alongside aging in our population of patients with thalassemia [39]. Abdominal ultrasonography is a routine examination in our patients with thalassemia and therefore all radiologic findings at US were for the first time evaluated simultaneously and correlated to several demographic, clinical, and laboratory data. Our findings confirmed previous evidence that stones and cysts are frequent among our populations [26,39]. Renal stones are a recognized complication in patients with thalassemia and have a multifactorial pathogenesis which involves the presence of hypercalciuria, hyperoxaluria, hypocitraturia, and hyperuricosuria, and is differently associated with tubular dysfunction linked mostly to excessive iron deposition in the renal tubules [8,40,41,42].

In agreement with a previous observation [43], renal stone formation involved more frequently splenectomized patients. In that series, encompassing only patients with thalassemia intermedia, potential underlying mechanisms in splenectomized patients that might contribute to stone formation were the significantly higher levels of average urate and nucleated red blood cells (NRBCs). In our current series, data on NRBCs were not available, and a significantly higher occurrence of nephrolithiasis was detected in patients with TM compared with those with TI. The role of splenectomy seems not to be related to uric acid, whose levels were comparable between patients with and without renal stones, suggesting both the presence of a suppressive effect of regular transfusions on erythopoietic activity and uric acid generation [44], and the higher use in TM of Deferasirox, a chelator with documented activity in uric acid reduction [45]. Differently, splenectomy, whose rate is decreasing particularly in patients with TI because of the early and late occurrence of complications [46,47], may simply mark the presence of more severe forms of thalassemia which are more prone to renal stone formation [48,49]. However, differently from that report, a significantly higher occurrence of nephrolithiasis was detected in patients with higher blood consumption and signs of renal impairment such as increased creatinine level and proteinuria. This finding suggests that renal iron overload and the related tubular dysfunction may be the pivotal contributors in renal stone formation. Further studies should be carried out to evaluate if renal iron deposition may correlate with renal stone formation. 

Our data regarding prevalence of renal cysts at the baseline and detection rate during the observation time are fully in agreement with a previous report exploring the prevalence of extracardiac findings encompassing renal cysts at MRI evaluation [39]. At baseline observation, patients with cysts were older and aging emerged as the independent predictor for further development of renal cysts. More interestingly, our longitudinal retrospective evaluation highlighted in a quite detailed way that renal cysts underwent evolution not only in terms of size but also in terms of Bosniak classification. Both the occurrence of a new cist and its progression were more frequently observed among patients with increased age and BMI and hypertension. Overall, our data on renal cyst progression indicate an increased risk compared to that observed in the general population [50], suggesting the need for accurate monitoring among our population once a cyst is identified. 

In our thalassemic population, the detection of 10 cases of RCC, all asymptomatic, corresponding to a crude incidence rate of 75.9 cases per 100,000 people, seems to be the most relevant finding of this report. It is well worth noting that 30% of these cases came from the canceration of a renal cyst, which usually is a small-probability event [51,52]. Bearing in mind that RCC has an incidence of 18.5 cases per 100,000 people in Italy, with a cumulative risk of 1.01 and with a peak incidence occurring between 60 and 70 years of age [53], the findings of the present study confirm our previous observation [29] and again suggest the presence of a particular renal cancer risk among our thalassemic patients, mainly among those with thalassemia major. This finding contrasts with a recent survey collecting data from eight Italian specialized centers where the incidence of malignant neoplasms in hemoglobinopathies as well as their sites and features were evaluated. The researchers found that the liver was the most common site of tumors in both sexes, showing a higher incidence (190 cases per 100,000 person-years) compared to the general population, but they did not find an increased incidence of RCC [54]. Furthermore, there is no evidence that during our observation period, a regional risk factor may have influenced this particular incidence, as data from the regional/local registry showed a slight reduced incidence as compared with those coming from the national registry [55].

In the general population, the most solid risk factors of RCC are age [53], gender [53], ethnicity [56], a history of smoking [57,58], hypertension [58,59], and obesity [60]. In the general population, there is a 1.5:1 male predominance [53]. The observed inverted gender relationship in our series is remarkable, but remains unexplained. Despite other risk factors being occasionally present among our patients, all were positive for HCV antibodies. One patient had chronic HCV infection while the remaining nine patients had eradicated the virus spontaneously (N = 5) or after the treatment with antiviral therapy (N = 4). Moreover, iron overload indices showed hepatic iron overload and/or a ferritin level ≥1000 ng/mL. In thalassemic patients, chronic HCV infection has been linked to several extrahepatic diseases [61,62,63] and, according to a systematic review and meta-analysis, in the general population, the risk of renal disease was significantly higher in subjects with positive HCV serologic status compared to non-infected patients [64]. Moreover, HCV infection has also been directly associated with an increased risk of renal-cell carcinoma, as observed in several epidemiologic studies [65,66] and confirmed in a subsequent meta-analysis [67]. Therefore, in our series, iron overload and previous HCV infection may have represented important oncogenic players that may have driven both renal carcinogenesis and the canceration of cysts. Notably, two cases were also affected by HCC. On the whole, these data further reinforce the need for both early HCV clearance and iron chelation in patients with thalassemia and HCV infection. Similarly, it is conceivable that in patients with present and past HCV infection, the US surveillance based on liver semestral abdominal ultrasound should also encompass the evaluation of renal cyst status. Further studies are needed to confirm this hypothesis, and the rate of HCV infection in the population evaluated for the estimate of RCC incidence should always be investigated. 

### Limitations

This study is limited by its retrospective design and single-center approach. Since our patients lived in a single, limited urban area (Naples), this cohort cannot be considered representative of the entire Italian thalassemic population. Our study had an exploratory nature and no family-wise error rate adjustments were performed. Larger, multicenter studies are needed to confirm and increase the generalizability of our findings.

## 5. Conclusions

Currently, abdominal US examinations are frequently requested in patients with thalassemia mainly for surveillance for the risk of HCC in patients with previous/contemporary HCV infection. Data from this report indicate that patients with thalassemia are significantly affected by asymptomatic renal diseases such as lithiasis, cysts, and cancer, which, in most cases, are detected incidentally by investigating various non-specific symptoms and other abdominal diseases, and are poorly associated with renal impairment and/or with biochemical markers. Further prospective studies are needed to better define the incidence and risk factors of these “incidentalomas”, that likely could not be considered an unwanted “side-effect” of imaging, but a real complication of thalassemia which would merit regular screening by US. Evidently, there is a need for the assessment of the performance of abdominal US in detecting renal disease and of the relative cost for the healthcare system, but several benefits in terms of the early diagnosis of renal diseases, patient outcomes, and the possibility to establish more precise guidelines for screening and monitoring in thalassemia patients are likely expected.

## Figures and Tables

**Table 1:** Baseline demographic, clinical, and instrumental findings in all thalassemia patients and in patients stratified according to the formation of renal stones during the follow-up.

	All Patients (N = 231)	No Renal Stones (N = 196)	Renal Stones (N = 35)	*p*-Value
Female sex, N (%)	132 (57.1)	112 (57.1)	20 (57.1)	1.000
Age (years)	36.18 ± 13.84	35.67 ± 14.31	39.03 ± 10.53	0.215
TM, N (%)	112 (48.5)	88 (44.9)	24 (68.6)	0.010
Annual blood consumption (mg/k die)	0.25 ± 0.53	0.19 ± 0.19	0.51 ± 1.24	0.005
Splenectomy, N (%)	95 (41.1)	73 (37.2)	22 (62.9)	0.005
Active or past HCV infection, N (%)	99 (42.9)	81 (41.3)	18 (51.4)	0.266
Hypertension, N (%)	16 (6.9)	14 (7.1)	2 (5.7)	0.759
Diabetes, N (%)	8 (3.5)	6 (3.1)	2 (5.7)	0.348
Body mass index (kg/m^2^)	23.19 ± 3.67	23.15 ± 3.81	23.45 ± 2.81	0.649
Serum ferritin (ng/mL)	1178.61 ± 1426.83	1185.39 ± 1497.15	1140.86 ± 959.63	0.327
Serum hemoglobin (g/dL)	9.67 ± 0.86	9.68 ± 0.84	9.60 ± 0.98	0.567
Serum transferrin receptor-1 (mg/L)	5.34 ± 2.95	5.51 ± 3.02	4.39 ± 2.32	0.021
Serum creatinine (mg/dL)	0.69 ± 0.31	0.69 ± 0.33	0.73 ± 0.15	0.014
Uric acid (mg/dL)	4.46 ± 1.27	4.45 ± 1.28	4.51 ± 1.24	0.726
Proteinuria (mg/dL)	17.21 ± 42.70	13.42 ± 23.15	38.12 ± 92.83	0.003
MRI LIC (mg/g dw)	6.01 ± 6.39	6.28 ± 6.62	5.73 ± 5.12	0.328

N = number; TM = thalassemia major; HVC = hepatitis C virus; MRI = magnetic resonance imaging; LIC = liver iron concentration.

**Table 2:** Results of univariate and Cox regression analysis for predictors of renal stones.

	Univariate Analysis	
	HR (95% CI)	*p*-Value
Female sex	0.96 (0.49–1.88)	0.913
Age	1.02 (0.99–1.04)	0.137
TM	2.26 (1.11–4.62)	0.025
Annual blood consumption	2.24 (1.53–3.27)	<0.0001
Splenectomy	2.57 (1.29–5.09)	0.007
Active or past HCV infection	1.37 (0.71–2.66)	0.351
Hypertension	0.87 (0.21–3.62)	0.845
Diabetes	1.73 (0.41–7.19)	0.453
Body mass index	1.02 (0.93–1.11)	0.745
Serum ferritin	1.00 (1.00–1.00)	0.818
Serum hemoglobin	0.89 (0.60–1.32)	0.566
Serum transferrin receptor-1	0.86 (0.74–1.00)	0.055
Serum creatinine	1.33 (0.66–2.68)	0.420
Uric acid	1.06 (0.82–1.37)	0.662
Proteinuria	1.01 (1.00–1.01)	0.004
MRI LIC	0.95 (0.89–1.03)	0.212

HR = hazard ratio; CI = confidence intervals; TM = thalassemia major; HVC = hepatitis C virus; MRI = magnetic resonance imaging; LIC = liver iron concentration.

**Table 3:** Baseline demographic, clinical, and instrumental findings in all thalassemia patients and in patients stratified according to the development of renal cysts during the follow-up.

	All Patients (N = 217)	No Renal Cysts (N = 177)	Renal Cysts (N = 40)	*p*-Value
Female sex, N (%)	127 (58.5)	101 (57.1)	26 (65.0)	0.357
Age (years)	35.17 ± 13.39	33.95 ± 13.35	40.55 ± 12.39	0.006
TM, N (%)	106 (48.8)	85 (48.0)	21 (52.5)	0.609
Annual blood consumption (mg/k die)	0.25 ± 0.54	0.21 ± 0.20	0.39 ± 1.18	0.845
Splenectomy, N (%)	82 (37.8)	62 (35.0)	20 (50.0)	0.078
Active or past HCV infection, N (%)	84 (38.7)	65 (36.7)	19 (47.5)	0.206
Hypertension, N (%)	10 (4.6)	6 (3.4)	4 (10.0)	0.072
Diabetes, N (%)	6 (2.8)	5 (2.8)	1 (2.5)	0.910
Body mass index (kg/m^2^)	23.03 ± 3.59	22.78 ± 3.64	24.16 ± 3.19	0.011
Serum ferritin (ng/mL)	1175.43 ± 1444.44	1225.41 ± 1524.97	955.51 ± 1002.96	0.741
Serum hemoglobin (g/dL)	9.64 ± 0.89	9.65 ± 0.93	9.61 ± 0.74	0.838
Serum transferrin receptor-1 (mg/L)	5.36 ± 2.91	5.38 ± 3.01	5.29 ± 2.40	0.711
Serum creatinine (mg/dL)	0.69 ± 0.31	0.69 ± 0.34	0.73 ± 0.18	0.034
Uric acid (mg/dL)	4.54 ± 1.28	4.55 ± 1.29	4.49 ± 1.25	0.810
Proteinuria (mg/dL)	17.49 ± 43.89	16.55 ± 47.13	21.60 ± 25.46	0.017
MRI LIC (mg/g dw)	6.28 ± 6.56	6.38 ± 6.72	5.89 ± 5.98	0.661

N = number; TM = thalassemia major; HVC = hepatitis C virus; MRI = magnetic resonance imaging; LIC = liver iron concentration.

**Table 4:** Results of univariate and Cox regression analysis for predictors of renal cysts.

	Univariate Analysis	
	HR (95% CI)	*p*-Value
Female sex	1.36 (0.71–2.60)	0.355
Age	1.04 (1.02–1.07)	0.001
TM	0.94 (0.50–1.74)	0.837
Annual blood consumption	1.26 (0.96–1.67)	0.102
Splenectomy	1.72 (0.92–3.19)	0.087
Active or past HCV infection	1.46 (0.78–2.71)	0.235
Hypertension	3.56 (1.26–10.05)	0.016
Diabetes	0.90 (0.12–6.57)	0.919
Body mass index	1.09 (1.01–1.18)	0.031
Serum ferritin	1.00 (1.00–1.00)	0.287
Serum hemoglobin	0.95 (0.67–1.34)	0.761
Serum transferrin receptor-1	1.00 (0.89–1.13)	0.959
Serum creatinine	1.27 (0.64–2.51)	0.490
Uric acid	0.98 (0.77–1.24)	0.844
Proteinuria	1.00 (0.99–1.01)	0.562
MRI LIC	0.99 (0.93–1.05)	0.684

HR = hazard ratio; CI = confidence intervals; TM = thalassemia major; HVC = hepatitis C virus; MRI = magnetic resonance imaging; LIC = liver iron concentration.

**Table 5:** Thalassemia patients who developed RCC during their lifetime: demographic, clinical, hematochemical, and instrumental characteristics at the time of diagnosis.

	Pt 1	Pt 2	Pt 3	Pt 4	Pt 5	Pt 6	Pt 7	Pt 8	Pt 9	Pt 10
RCC subtype	clear-cell	mucinous tubular and spindle-cell	clear-cell	clear-cell	clear-cell	clear-cell	chromophobe	clear-cell	clear-cell	clear-cell
RCC staging	T1a NX	T1b N0	T3b	T2 N1	not available	T3a	T1 N0	not available	T1a	not available
RCC stage group	I	not available	3	2	1	not available	1	not available	II	not available
RCC site	right kidney	right kidney	right kidney	right kidney	right kidney	right kidney	right kidney	right kidney	left kidney	left kidney
Canceration of a previous renal cyst	no	yes	yes	no	no	no	no	no	no	yes
Symptoms	no	no	no	no	no	no	no	no	no	no
Co-presence of hepatic cell carcinoma	yes	no	no	no	no	yes	no	no	no	no
Disease	TI regularly transfused	TI non-transfused	TM	TM	TM	TM	TM	TM	TM	TM
Genotype (Phenotype)	CD39/IVS-1,6 (β0 β+)	CD39/IVS-1,6 (β0 β+)	IVS-1,6/IVS-1,110 (β+ β+)	CD39/IVS-1,6 (β0 β+)	IVS-1,110/IVS-1,110 (β+ β+)	CD39/IVS-1,1 (β0 β0)	IVS-1,6/IVS-1,6 (β+ β+)	CD6/IVS-2,1 (β0 β0)	IVS-1,1/IVS-1,1 (β0 β0)	CD39/IVS-1,110 (β0 β0)
Sex	female	female	female	male	female	male	female	male	female	male
Age at diagnosis (years)	60.4	54.3	39.0	44.6	53.1	44.9	53.9	50.7	41.5	42.0
Splenectomy	yes	yes	yes	yes	yes	no	yes	yes	yes	yes
Anti-HCV	positive	positive	positive	positive	positive	positive	positive	positive	positive	positive
HCV RNA	negativized after antiviral therapy	negative	negative	positive	negativized after antiviral therapy	negative	negativized after antiviral therapy	negative	negativized after antiviral therapy	negative
Smoking	never	never	never	never	never	never	never	yes	yes	never
Serum ferritin (ng/mL)	396.0	519.0	180.0	4020.0	1283.0	419.0	750.0	3800.0	803.0	250.0
Serum hemoglobin (g/dL)	10.8	8.6	9.5	10.3	8.8	9.5	10.4	9.7	9.8	10.5
Serum transferrin receptor-1 (mg/L)	3.8	8.3	not available	3.4	2.3	7.0	4.5	5.2	3.0	2.8
Serum creatinine (mg/dL)	0.7	0.6	not available	1.1	0.8	0.6	0.5	0.5	0.6	0.8
Uric acid (mg/dL)	3.0	6.4	not available	6.6	4.4	6.0	4.1	5.0	3.5	3.8
Proteinuria (mg/dL)	0.0	12.4	not available	7.8	7.7	8.5	0.0	0.0	22.2	35.9
MRI LIC (mg/g dw)	1.9	8.3	not available	4.9	9.9	3.3	1.1	11.3	5.9	14.8

Pt = patient; RCC = renal cell carcinoma; TI = thalassemia intermedia; TM = thalassemia major; HVC = hepatitis C virus; RNA = ribonucleic acid; MRI = magnetic resonance imaging; LIC = liver iron concentration.

## Data Availability

Data will be made available upon request to the corresponding author.
